# The Evaluation of Equine Allogeneic Tenogenic Primed Mesenchymal Stem Cells in a Surgically Induced Superficial Digital Flexor Tendon Lesion Model

**DOI:** 10.3389/fvets.2021.641441

**Published:** 2021-03-05

**Authors:** Eva Depuydt, Sarah Y. Broeckx, Lore Van Hecke, Koen Chiers, Leen Van Brantegem, Hans van Schie, Charlotte Beerts, Jan H. Spaas, Frederik Pille, Ann Martens

**Affiliations:** ^1^Global Stem cell Technology, Part of Boehringer Ingelheim, Evergem, Belgium; ^2^Department of Surgery and Anaesthesiology of Domestic Animals, Faculty of Veterinary Medicine, Ghent University, Merelbeke, Belgium; ^3^Department of Pathology, Bacteriology and Poultry Diseases, Faculty of Veterinary Medicine, Ghent University, Merelbeke, Belgium; ^4^Department of Clinical Sciences, Faculty of Veterinary Medicine, Utrecht University, Utrecht, Netherlands; ^5^Department of Research and Development, UTC Imaging, Stein, Netherlands; ^6^Department of Veterinary Medical Imaging and Small Animal Orthopaedics, Faculty of Veterinary Medicine, Ghent University, Merelbeke, Belgium

**Keywords:** mesenchymal stem cells, allogeneic, tenogenic primed, SDFT, peripheral blood, horse

## Abstract

**Background:** Tendon injuries are very common in horses and jeopardize the athletic performance, and due to the high risk of reinjury may lead to early retirement. The use of mesenchymal stem cells for the treatment of equine tendon disease is widely investigated because of their regenerative potential. The objective of this study is to investigate the safety and efficacy of equine allogeneic tenogenic primed mesenchymal stem cells (tpMSCs) for the management of tendinitis in horses.

**Methods:** A core lesion was surgically induced in the superficial digital flexor tendon of both forelimbs of eight horses. After 7 days, one forelimb was treated with tpMSCs, while the contralateral forelimb served as an intra-individual control and was treated with saline. A prescribed exercise program was started. All horses underwent a daily clinical evaluation throughout the entire study period of 112 days. Blood samples were taken at different time points for hematological and biochemical analysis. Tendon assessment, lameness examination, ultrasound assessment and ultrasound tissue characterization (UTC) were performed at regular time intervals. At the end of the study period, the superficial digital flexor tendons were evaluated macroscopically and histologically.

**Results:** No suspected or serious adverse events occurred during the entire study period. There was no difference in local effects including heat and pain to pressure between a single intralesional injection of allogeneic tpMSCs and a single intralesional injection with saline. A transient moderate local swelling was noted in the tpMSC treated limbs, which dissipated by day 11. Starting at a different time point depending on the parameter, a significant improvement was observed in the tpMSC treated limbs compared to the placebo for echogenicity score, fiber alignment score, anterior-posterior thickness of the tendon and echo type by UTC assessment. Immunohistochemistry 112 days post-injection revealed that the amount of collagen type I and Von Willebrand factor were significantly higher in the tendon tissue of the tpMSC group, while the amount of collagen type III and smooth muscle actin was significantly lower.

**Conclusion:** Equine allogeneic tenogenic primed mesenchymal stem cells were shown to be well-tolerated and may be effective for the management of tendon injuries.

## Introduction

Tendon injuries are very common in both human (1, 2) and equine athletes (3, 4), and often result in reinjury or early retirement (5). In racehorses, tendon and ligament injuries are the most common orthopedic diseases (6–8), but also dressage, eventing, show jumping, and pleasure riding horses suffer from tendon pathologies (9, 10). The formation of scar tissue as a sequel to tendon injury hampers the natural healing process and subsequently impairs the normal tendon function (11).

Healthy tendon tissue is mainly composed of type I collagen, contributing to a remarkable tensile strength and elasticity. In case of an acute tendon trauma, an inflammatory phase is initiated that is characterized by the formation of a local hematoma and induction of an inflammatory reaction at the site of the tendon tear or lesion (12). Around 7 days after the injury, the proliferative phase develops, which includes angiogenesis and fibroplasia with random depositions of collagen type III (13). The smaller and less organized fibrils of collagen type III result in loss of original tensile strength and elasticity (13, 14). Finally, 6–8 weeks after injury, the remodeling phase sets in, which is characterized by a decrease in type III collagen and an increase in synthesis of collagen type I. However, the repair tissue never reaches the tensile characteristics of healthy tendon, resulting in the formation of rigid scar tissue with increased risk of tendon reinjury (12, 15). Despite major progress in the early detection of tendon injuries and the development of advanced therapies, the healing process is slow with an inferior quality of the repaired tendon tissue (5). Therefore, there is a need for further research on treatments aiming to restore tendon functionality, encouraging the development of regenerative treatments such as cell-based therapies.

Recently, promising advances have been made in the treatment of equine tendon injuries in the field of mesenchymal stem cell (MSC) therapy. Naturally occurring superficial digital flexor tendinopathy treated with MSCs resulted in a reduced reinjury rate and better tendon echogenicity (5, 16–19). Additionally, experimental studies using MSCs as treatment for surgically or enzymatically induced tendon core lesions report better tendon echogenicity and a higher collagen type I/collagen type III ratio (20, 21). Although MSCs are a valuable option in the treatment of tendon disease, their tenogenic properties and regenerative effects could be compromised by the inflammatory environment of the acute tendon injury (22). This was observed in the study of Geburek et al. (23) where no significant reduction in clinical signs of inflammation and no reduction of fluid during ultrasonography could be detected following adipose tissue-derived MSC (AT-MSC) injection in surgically induced superficial digital flexor tendon (SDFT) core lesions. Although not in horses, ectopic bone formation has been reported in rabbits and mice after administration of bone marrow-derived MSCs (BM-MSCs) into acute tendon lesions (14, 24–26). In order to avoid these disadvantageous effects, MSCs can be tenogenic primed before clinical application (27). Previous work reported the safe use of equine allogeneic peripheral blood-derived tenogenic primed MSCs (tpMSCs) in combination with platelet rich plasma (PRP) and positive outcome in horses with naturally occurring tendinopathies (28, 29). Broeckx et al. (28) described the successful treatment of lesions in the suspensory ligament and SDFT with tpMSCs in combination with PRP in 24 out of 25 horses based on ultrasonographic and lameness evaluations 6 weeks post-treatment. Moreover, Beerts et al. (29) reported that 85.7% of horses with a naturally occurring lesion in the SDFT returned to their previous performance level after treatment with tpMSCs in combination with PRP in a 2-year follow-up study. These findings indicate that the use of tpMSCs in combination with PRP is promising for future applications. However, placebo-controlled, randomized and blinded studies to objectively investigate the safety and efficacy of allogeneic tpMSCs are currently lacking.

Therefore, the goal of this study was to assess the safety and efficacy of a single intralesional injection of equine allogeneic tenogenic primed MSCs in a surgical induced core lesion model in the equine SDFT. In this research, the treatment effect of tpMSCs was compared to a control product (saline) evaluating tendon and lameness assessment, echogenicity and fiber alignment scoring, ultrasonographic tissue characterization and immunohistochemistry data.

## Materials and Methods

This animal study (EC_2017_001) including the blood collection from the donor horses (EC_2016_003) was approved by an independent ethics committee approved by the Flemish government (permit number: LA1700607). The study was conducted under GLP standards in accordance with national and international animal welfare regulations (Directive 2001/82/EC as amended, Belgian animal welfare legislation (KB 29/05/2013), Directive 2010/63/EU and EMEA/CVMP/816/00-Final).

### Study Design and Horses

Eight healthy warmblood horses, four geldings and four mares (age ranged between 3 and 12 years) were enrolled in this blinded, placebo-controlled, randomized study. The horses were free of lameness and swelling, heat or reaction to palpation of the SDFT of both forelimbs at the time of enrollment. To reduce the number of horses, this study compared the effect of the investigational product (IVP) and the control product (CP) within the same horse. Each of the horses received the CP in one of its forelimbs and the IVP in its contralateral forelimb. The treatment allocation was randomized. Thus, one forelimb within a horse was randomly allocated to the IVP group (*n* = 8), while the contralateral limb was assigned to the CP group (*n* = 8). All clinical, tendon, lameness, ultrasound, and histopathological parameters were evaluated by an examiner blinded to the treatment.

### Investigational Product and Control Product

The IVP consisted of a proprietary formulation of equine allogeneic tenogenic primed mesenchymal stem cells (tpMSCs) prepared from peripheral blood as previously described (28). Briefly, blood was collected aseptically from the *vena jugularis* in sterile ethylenediaminetetraacetic acid (EDTA) tubes. After gradient centrifugation, the interphase was isolated, seeded and cultivated in culture medium as previously described (30). At 80% confluency, the cells were trypsinized and cultured in tenogenic priming medium. At the next confluency, the cells were trypsinized and resuspended in 1 mL Dulbecco's modified Eagle medium low glucose supplemented with 10% dimethyl sulfoxide. The samples were cryopreserved at −80°C until further use. In the present study, two different tpMSC batches of the same donor horse that were produced on two different time points were used. Each forelimb within one horse was randomly allocated either to one of the two batches. The donor horse was screened for equine pathogens as previously reported by our group (28).

The control product consisted of 1 mL sterile saline injectable solution (0.9% NaCl).

### Surgical Procedure and Post-operative Treatment

One week before treatment administration [day-7: reported time point that fibroblast ingrowth starts (13)], a standardized lesion in the SDFT of both forelimbs was created using a surgical model adapted from Schramme et al. (31) and Bosch et al. (32, 33) (Table 1). The surgery was performed under general anesthesia and followed routine clinical procedures. Pre-operatively, horses were sedated using romifidine (0.8 mL/100 kg body weight; Sedivet®, intravenous). General anesthesia was induced using ketamine (2.2 mL/100 kg body weight; Ketamidor®, intravenous) and midazolam (0.1 mg/kg body weight; Midazolam Mylan, intravenous). Anesthesia was maintained during the surgical procedure by administration of isoflurane (IsoFlo® MAC^*^ = 1.3%) under controlled ventilation. The horses were positioned in right lateral recumbency. After circumferential clipping and aseptical preparation of the surgical sites, a small skin incision (1 cm) was made in the palmar midline of the limbs just proximal to the digital flexor tendon sheath. Through a small longitudinal incision into the SDFT (1 cm), a 3.5 mm Steinmann pin was inserted in the core of the SDFT under ultrasonographic guidance and a central lesion over an area of 8 cm was created. Next, an arthroscopic burr (∅ 3.5 mm Razorcut blade; C9110; Linvatec, Largo, Florida, USA) was inserted in the defect created by the Steinmann pin and was advanced proximally over a length of 8 cm. After activation, the burr was slowly retracted over 1 min while performing a rotating movement making sure that the medial, palmar and lateral aspect of the tendon was debrided. Local pressure on the SDFT was exerted to assist in this debridement. The shaver was used in forward modus at 5,000 rpm. No suction was applied. The incisions in the paratenon and skin were sutured using simple interrupted sutures with resorbable polyglyconate (Monosyn USP 3-0 B. Braun).

**Table 1:** Comparison of surgically induced SDFT core lesions in the current study with the model according to Schramme et al. (31) and Bosch et al. (32, 33).

	**Present study**	**Schramme et al. (31)**	**Bosch et al. (32, 33)**
Instrument	Arthroscopic burr	Synovial resector	Arthroscopic burr
Lesion diameter	3.5 mm	3.5 mm	3.5 mm
Lesion length	8 cm	8 cm	7 cm
Suction	No	Yes	No
Ultrasonographic guidance	Yes	Yes	No
Bandage	7 days	Limbs were bandaged after surgery, period of bandaging not reported	14 days
Treatment	tpMSCs	Not applicable	PRP
Start exercise scheme	1 week after surgery	3 weeks after surgery	4 weeks after surgery
Follow-up	16 weeks	2, 4, 8, and 12 weeks	24 weeks

A bandage was applied to the limbs for 7 days after surgery, until treatment administration. A new bandage was placed after treatment (day 0). The bandage was removed for tendon assessment on day 1–3 after treatment and replaced after the assessment. From day 4 to 7 after treatment, the bandage was removed at 10 a.m. ± 1 h and replaced at 4 p.m. ± 1 h right after tendon assessment. One week after surgery, the sutures and the bandage were removed permanently.

Horses were walked for 6 days after surgery using a horse walker (Table 2) and received an intralesional tpMSC or placebo treatment at 7 days post-surgery as described below. A gradually increasing exercise protocol was started from day 4 after treatment onwards using a horse walker (Table 2). Care was taken to exercise in both directions equally.

**Table 2:** Exercise protocol of the horses after bilateral surgical induction of a SDFT core lesion.

**Time point**	**′W = minutes walking, once per day**						
	**′T = minutes trotting, once per day**						
Day −7	Stall rest						
Day −6 to −5	2.5**′** W			Change direction	2.5**′** W		
Day −4 to −1	5**′** W			Change direction	5**′** W		
Day 0	Stall rest						
Day 1–3	Stall rest						
Day 4–14	2.5**′** W			Change direction	2.5**′** W		
Week 3	5**′** W			Change direction	5**′** W		
Week 4	7.5′ W			Change direction	7.5′ W		
Week 5	10′ W			Change direction	10′ W		
Week 6	12.5′ W			Change direction	12.5′ W		
Week 7	15′ W			Change direction	15′ W		
Week 8–9	5′ W	1′ T	5′ W	Change direction	5′ W	1′ T	5′ W
Week 10–11	5′ W	2′ T	5′ W	Change direction	5′ W	2′ T	5′ W
Week 12-13	5′ W	3′ T	5′ W	Change direction	5′ W	3′ T	5′ W
Week 14–15	5′ W	4′ T	5′ W	Change direction	5′ W	4′ T	5′ W
Week 16	5′ W	5′ T	5′ W	Change direction	5′ W	5′ T	5′ W

*This exercise scheme was performed once a day. Care was taken to exercise equally in both directions and time was divided equally over both sides. ′W = minutes walking; ′T = minutes trotting*.

### Treatment Administration

At day 0, one week post-surgery, the horses were mildly sedated with detomidine hydrochloride (0.1 mL/100 kg body weight, Detogesic®, intravenous). The area around the injection site was clipped and aseptically prepared. The tendon was punctured laterally and the treatments (IVP and CP) were administered by a single intralesional injection in the SDFT core lesion under ultrasonographic guidance to ensure a successful administration to the site of the lesion. For the IVP, the tpMSCs were thawed in the palm of a hand and drawn into a syringe. Both IVP and CP treatments were administered by an assigned veterinarian (the dispenser), who did not participate in any of the clinical or laboratory examinations. This way blinding of the personnel who assessed the efficacy and safety parameters and performed the post-mortem evaluation was ensured.

### Clinical Assessment

A general clinical examination of the horses was conducted and recorded daily throughout the study (from day −10 until day 112 ± 1). The body temperature, heart and respiratory rates, mucous membrane color, capillary refill time, and feed intake were evaluated. The body weight of the horses was recorded on day −10 and 112 ± 1.

During the study period from the day of treatment administration (day 0) to study completion, all horses were observed daily for adverse events, serious adverse events and suspected adverse drug reactions and these were recorded when occurring.

### Blood Sample Collection and Analysis

Blood was collected from the vena jugularis into EDTA tubes and serum clot activator tubes for analysis of following hematological and biochemical blood parameters in an ISO15189 certified laboratory (Vebio, France): white blood cell count, red blood cell count, hemoglobin, hematocrit, mean corpuscular volume, mean corpuscular hemoglobin, mean corpuscular hemoglobin concentration, neutrophils, eosinophils, lymphocytes, platelet count, basophils, monocytes, reticulocytes, blood urea nitrogen, urea, creatinine, total protein, albumin, globulin, alkaline phosphatase, serum glutamic oxaloacetic transaminase, total and direct bilirubin, creatinine kinase, lactic dehydrogenase, and gamma-glutamyl transpeptidase.

### Tendon Assessment

The tendon assessment was performed on following days: −9 (before surgical procedure), 0 (before treatment), daily on days 1–14, 28, 42, 56, 84, and 112 ± 1. Both forelimbs of each horse were evaluated at each time point. A score was given to the local heat, pain to pressure, and the swelling (Table 3). Also, the circumference measurement (cm) of each forelimb was recorded using a measuring tape. The tape was placed at the level of the metacarpus just proximal to the digital flexor tendon sheath with the limb in a weight bearing position. All measurements were performed on both forelimbs and the values at each time point were compared to the baseline values (day −9 and day 0).

**Table 3:** Clinical scores used in all horses to assess local heat, swelling, and pain to pressure.

**Parameter**	**Score system**	**Definition**
Heat	0	No increased temperature sensation
	1	Slightly increased temperature sensation
	2	Moderately increased temperature sensation
	3	Severely increased temperature sensation
Pain to pressure	0	No pain to pressure
	1	Slight pain to pressure
	2	Moderate pain to pressure
	3	Severe pain to pressure
Swelling	0	No swelling
	1	Slight swelling
	2	Moderate swelling
	3	Severe swelling

### Lameness Assessment

Lameness was assessed on following days: −9 (before the surgical procedure), 0 (before treatment), 14, 56, and 112 ± 1 according to the American Association of Equine Practitioners (AAEP) scoring system (Table 4). The examining vet also evaluated whether the lameness was attributed to the left or right front limb or if the lameness was considered bilateral. The horses were examined by walking and trotting in hand, on a straight line and with a loose line to the halter. Evaluation in a circle was performed during the revalidation exercise in the horse walker on a soft surface, and on a lunge. A firm, non-slippery surface was used for trotting on a straight line and for lunging.

**Table 4:** AAEP scoring.

**Grade**	**Degree of lameness**
0	Lameness not perceptible under any circumstances
1	Lameness is difficult to observe and is not consistently apparent, regardless of circumstances
2	Lameness is difficult to observe when walking or trotting in a straight line, but consistently apparent under certain circumstances
3	Lameness is consistently observable at a trot under all circumstances
4	Lameness is obvious at walk
5	Lameness produces minimal weight bearing in motion and/or at rest or a complete inability to move

### Ultrasound Tissue Characterization

The structural integrity of the SDFT of both forelimbs of each horse was qualified using established protocols for an ultrasound tissue source characterization (UTC) (32, 34). The UTC assessment was performed on the following days: −9 (before surgery), 0 (before treatment), 14, 28, 42, 84, and 112 ± 1. The horses were mildly sedated with detomidine hydrochloride (0.1 cc/100 kg body weight, Detogesic®, intravenous) and both forelimbs were clipped, shaved and washed at the palmar aspect of the metacarpus prior to each scan. Coupling gel was applied to ensure maximum contact. For the UTC examination, a 12-5 MHz multi-frequency ultrasound probe (12 L5; Terason Ultrasound, Teratech Corporation, Burlington, MA, USA) connected to a laptop computer (MacBook Pro® 17 inch, Apple, Cupertine, CA, USA) loaded with software for data acquisition and analysis (UTC^TM^ Software V.1.0.1 2010, UTC Imaging, Stein, The Netherlands) was used. The probe was fixed in a motorized tracking device with built-in standoff pad (UTC-Tracker^TM^; UTC Imaging). Settings (depth, gain, focal zone) were standardized and all examinations were performed by the same operator with the horse in weight bearing position, equally on both forelimbs. With the help of the tracking device, the probe moved automatically from proximal to distal parallel to the long axis of the tendon at constant speed over a distance of 12 cm. Compilation of contiguous transverse images was conducted at precise steps of 0.2 mm, including the insertion point of the arthroscopic burr (scar in the epitenon) as the reference point for the analysis. A clear ultrasound image of the SDFT was obtained and checked to ensure no artifacts were present due to movement or poor contact. The compiled 600 transverse ultrasound images were reconstructed into a three-dimensional data block of ultrasound information and stored digitally until the end of the examination period. The stability of the echo pattern of corresponding pixels in contiguous transverse images can be analyzed (UTC2011® Analyser V1.0.1; UTC Imaging; window size 17). Four different pixel types were classified based on their stability and degree of persistence, resulting in four echo types related to stages of ultrastructural integrity (Table 5) (34). In the analysis, a 4 cm long tendon segment from 2 to 6 cm proximal to the scar in the epitenon was selected. In this selected segment, the percentage of echo types generated by the tendon structure was evaluated.

**Table 5:** Echo types and corresponding colors that were discriminated and determined throughout the study (34).

**Echo type (color code)**	**Echo source according to histology**
Echo type I (green)	Echo generated by intact and fully aligned fiber bundles
Echo type II (blue)	Echo generated by discontinuous and less aligned fiber bundles
Echo type III (red)	Echo generated by a mainly fibrillar matrix with accumulation of collagen fibrils not (yet) organized into fiber bundles
Echo type IV (black)	Echo generated by an amorphous matrix and/or fluid

### Ultrasound Assessment

The ultrasound images obtained with the UTC device were used for the ultrasound assessment. During this evaluation, three different parameters were evaluated. First, the echogenicity of each lesion was graded according to a pre-defined scoring system (Table 6). Second, the fiber alignment was graded according to the estimated percentage of parallel fiber bundles in the lesion (Table 6). Third, an increase in anterior-posterior thickness (APT) in cm was recorded to monitor tendon repair.

**Table 6:** Ultrasound assessment.

**Parameter**	**Score system**	**Definition**
Echogenicity	0	Normal echogenicity
	1	Mildly hypoechoic
	2	Moderate hypoechoicity
	3	Severe hypoechoicity
Fiber alignment score	0	≥75% parallel fiber bundles in the lesion
	1	50–74% parallel fiber bundles in the lesion
	2	25–49% parallel fiber bundles in the lesion
	3	≤25% parallel fiber bundles in the lesion

### Histological Sampling Procedure

At day 112 ± 1 of the study, the horses were humanely euthanized after sedation with romifidine (0.8 mL/100 kg body weight; Sedivet®) using ketamine (2.2 mL/100 kg body weight; Ketamidor®), midazolam (0.1 mg/kg body weight; Midazolam Mylan) and T61 (0.3 mL/kg body weight; Hoechst Roussell Vet, Frankfurt, Germany).

Immediately after euthanasia, the SDFT and paratenon of both forelimbs of each horse were collected. After closing the surgical wound the skin remained slightly raised, leaving the original entry point of the arthroscopic burr visible throughout the entire study period. This remaining scar could be used as a reference for the entry point of the burr. The tendon segment located 12 cm proximal to the entrance of the burr was obtained. Next, transversal and longitudinal tendon sections were excised, and a macroscopic evaluation of these sections was performed. Finally, digital photographs were taken from each tendon before fixation in 10% natural-buffered formalin.

Samples of the cubital lymph nodes, prescapular lymph nodes, spleen, liver, brain, kidneys, adrenal glands, lungs, heart (ventricles and septum), stomach, duodenum, jejunum, ileum, colon, and cecum were fixed in 10% natural-buffered formalin for further histologic evaluation.

### Macroscopic and Histopathologic Examination

A macroscopic evaluation of tendon, paratenon, and cubital lymph nodes of both forelimbs of each horse was performed. The lesion and color of the tendon were observed and recorded (lesion visible or not, discoloration visible or not). The paratenon and lymph nodes were evaluated for abnormalities of the thickness and color. Normal thickness and color were differentiated from any other abnormalities. Any abnormality found was recorded.

Histological sections were prepared from all formalin-fixed samples and stained with haematoxylin & eosin (HE). All samples were evaluated for presence of ectopic tissue and presence of inflammation.

Samples of tendon and paratenon were scored according to the general accepted semi-quantitative score system of Movin et al. (35). Briefly, fiber structure, fiber arrangement, roundness of the nuclei, regional variations in cellularity, vascularity, collagen stainability, glycosaminoglycan content, and the presence of inflammatory cells were evaluated by an experienced pathologist (score 0: normal; score 1: slightly abnormal; score 2: moderately abnormal; score 3: markedly abnormal).

### Immunohistochemistry

Immunohistochemistry was performed to evaluate the distribution percentage of collagen type I (COL I), collagen type III (COL III), Von Willebrand Factor (VWF) and smooth muscle actin (SMA). For each staining, 5 μm thick tendon sections were cut and mounted on Thermo Scientific SuperFrost Plus^TM^ adhesion slides (Fischer Scientific, UK). Next, slides were deparaffinized and rehydrated in xylene and decreasing concentrations of alcohol in H_2_O (96, 80, 70, 60, and 100% H_2_O).

### Collagen Type I

Antigen retrieval was performed by incubation with pronase (1 mg/mL) and subsequently hyaluronidase (10 mg/mL) at 37°C for 20 min. Sections were treated with goat serum [10% in phosphate-buffered saline (PBS)] for 15 min at room temperature, after which they were incubated with the primary polyclonal rabbit collagen type I antibody (1:500; ab138492; Abcam) for 60 min at room temperature. Next, the secondary anti-rabbit antibody (EnVision horseradish peroxidase (HRP) anti-rabbit; K4001; Agilent) was incubated for 30 min at room temperature in the dark. Visualization was performed in a 3.3-diaminobenzidine solution (DAB; K3468; Agilent) for 7 min at room temperature in the dark. The cell nuclei were counterstained with hematoxylin, after which the sections were dehydrated and coverslips were applied. In between all steps, the sections were washed extensively and repeatedly with 0.1% PBS-Tween 20. Only after incubation with the secondary HRP antibody, the slides were rinsed with PBS instead of 0.1% PBS-Tween 20.

### Collagen Type III

Antigen retrieval was performed using an EDTA buffer (10% in distilled water; pH 9.0) for 30 min at 95°C. Next, slides were incubated with H_2_O_2_ (S202386-2; Agilent) for 5 min at room temperature in the dark followed by a treatment with normal goat serum (30% in PBS) for 30 min at room temperature. Subsequently, incubation with the primary polyclonal rabbit collagen type III antibody (1:200; ab7778; Abcam) was performed for 90 min at room temperature followed by a secondary incubation with anti-rabbit antibody (biotinylated anti-rabbit; E0432; Agilent) for 30 min at room temperature in the dark. Next, incubation with ABC-HRP (Vector PK-6100) took place for 30 min in the dark at room temperature. Visualization was performed in DAB as described above.

### Von Willebrand Factor

Slides were incubated with proteinase K (0.05M in Tris-HCL, pH 7.6; S3004; Agilent) for 15 min at room temperature for antigen retrieval. Next, sections were incubated with H_2_O_2_ (S202386-2; Agilent) for 5 min followed by the primary polyclonal rabbit VWF antibody (1:3200; A0082; Agilent) incubation for 30 min at room temperature. Subsequently, the secondary anti-rabbit antibody (EnVision HRP anti-rabbit; K4003; Agilent) was incubated for 30 min at room temperature in the dark. Visualization was performed as described above.

### Smooth Muscle Actin

Antigen retrieval was performed using a citrate buffer (10% in distilled water; pH 6.0) for 13 min in the microwave. Next, sections were incubated with H_2_O_2_ (S202386-2; Agilent) for 5 min followed by incubation with the primary monoclonal mouse SMA antibody (1:200; M0851; Agilent) for 30 min at room temperature. Then, the secondary anti-mouse antibody (EnVision HRP anti-rabbit; K4001; Agilent) was incubated for 30 min at room temperature in the dark. Visualization was performed as mentioned above.

Positive stainings were confirmed on microscopy. For each section, a randomly selected area was photographed (at ×40 magnification). The percentage of the total stained area (representing COL I, COL III, SMA, or VWF content) was calculated with software (LAS, version 4.1, Leica microsystems, Diegem, Belgium).

### Data Analysis

All statistical analyses were performed using SAS® statistical analysis software version 9.3. The groups were compared to baseline using ANOVA for the lameness assessment, increase of tendon circumference and increase of local pain to pressure. The treatment groups were compared using the Mantel-Haenszel test for tendon assessment (pain, heat, and swelling), lameness assessment, ultrasound assessment (echogenicity and fiber alignment score) and histopathology. Groups were compared using Wilcoxon test for tendon circumference, anterior-posterior thickness, UTC assessment and immunohistochemistry. Groups were compared using the Fisher's exact test for incidence of adverse events, serious adverse events and suspected adverse drug reactions, incidence of abnormal clinical signs and decrease of body weight. *P*-value ≤ 0.5 was considered statistically significant.

## Results

### Horses

Due to the study design each horse received the CP in one of its forelimbs, whereas, the contralateral limb in each horse was treated with the IVP.

### Clinical Assessment

The clinical examination parameters, namely body temperature, heart, and respiratory rates were all in the physiological range for all horses at all time points. There was a mean increase of body weight of 3.8 kg (± 4.05) from day −9 to day 111.

One adverse event was recorded: one horse showed mild signs of colic 38 days following treatment. The horse also had reduced feed intake the day after. No treatment was necessary and the horse fully recovered. The condition was deemed not to be related to the study medication.

No serious adverse events or suspected adverse drug reactions were observed throughout the study period.

### Hematological and Serum Biochemistry Parameters

The analyses of the hematological and serum biochemistry parameters revealed no serious deviations from the reference values (Supplementary Material). The observed deviations were marginally out of range and were regarded as not clinically relevant by the attending veterinarian.

### Tendon Assessment

#### Local Heat

**Figure 1:**
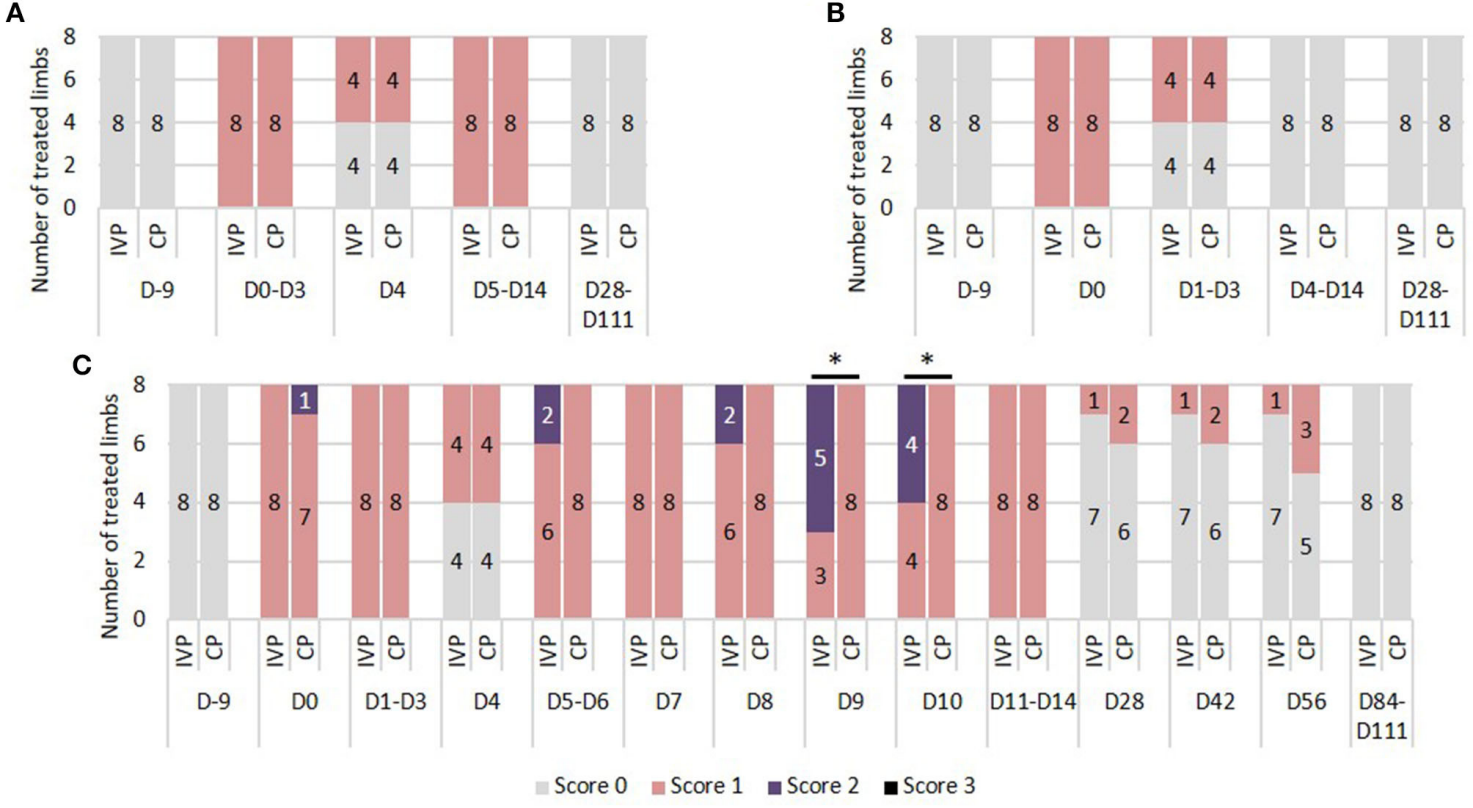
Tendon assessment. **(A)** Local heat. 0 = no increase in temperature sensation; 1 = slightly increased temperature sensation; 2 = moderately increased temperature sensation; 3 = severely increased temperature sensation. **(B)** Local pain to pressure. 0 = no pain to pressure; 1 = slight pain to pressure; 2 = moderate pain to pressure; 3 = severe pain to pressure. **(C)** Local swelling. 0 = no swelling; 1 = slight swelling; 2 = moderate swelling; 3 = severe swelling. ^*^P-values ≤ 0.05.

There were no differences between the IVP group and the CP group during the study period. Prior to surgery (day −9) and from day 28 onwards, none of the horses showed increase in local heat at the level of the SDFT area. On days 0–14 a slightly increased temperature was observed in all limbs, with the exception of day 4 where half of the limbs in each treatment group showed no increased temperature (Figure 1A).

#### Pain to Pressure

There were no differences in pain to pressure between the IVP and CP group. Prior to surgery (day −9) and from day 4 onwards, none of the horses showed local pain to pressure. Between day 1 and 3, no or slight pain to pressure was observed in half of the limbs in each treatment group (Figure 1B).

#### Local Swelling

On day −9, 84, and 111 none of the horses showed local swelling. On all other observation days, the scores ranged between 0 and 2. Significant differences between groups were seen on day 9 and 10, where the percentage of moderate swelling (score 2) was significantly higher in the IVP group compared to the CP group (*P* = 0.009 and *P* = 0.025, respectively) (Figure 1C).

No significant differences in *tendon circumference* could be observed between groups throughout the study duration. However, when changes of the mean tendon circumference were compared to the baseline values of day 0 (after surgery, but before treatment administration), a significant difference between the IVP treated limbs and the contralateral CP treated limbs could be determined on day 9 and 10 (*P* ≤ 0.05). The mean circumference of the CP group remained the same, whereas a mean increase of 0.2 cm was observed in the IVP group when compared to baseline.

**Figure 2:**
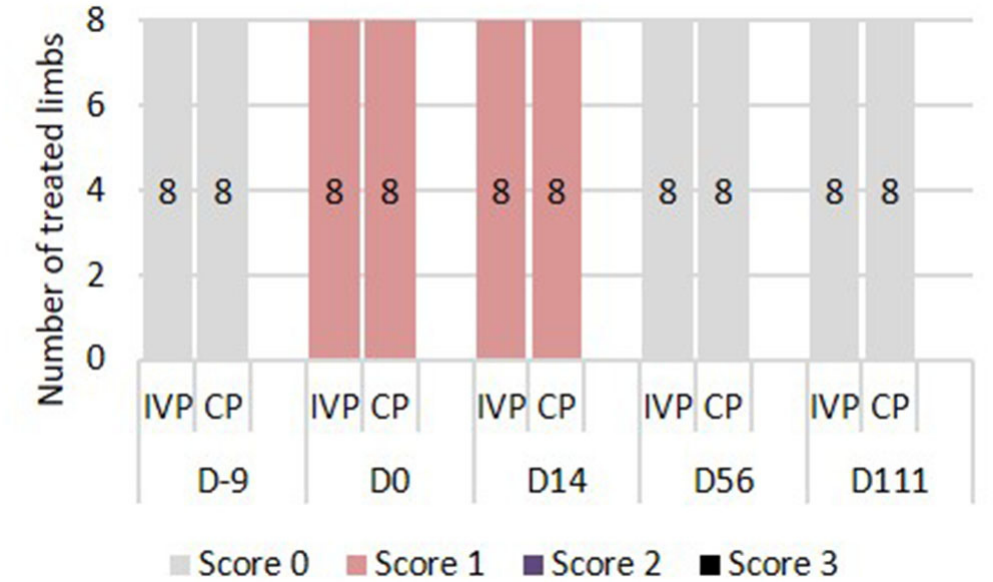
Lameness assessment. 0 = Lameness not perceptible under any circumstances; 1 = Lameness is difficult to observe and is not consistently apparent, regardless of circumstances; 2 = Lameness is difficult to observe at a walk, or when trotting in a straight line, but consistently apparent under certain circumstances; 3 = Lameness is consistently observable at a trot under all circumstances; 4 = Lameness is obvious at a walk 5 = Lameness produces minimal weight bearing in motion and/or at rest or a complete inability to move.

There was no significant difference in visual lameness scores between the two treatment groups (IVP vs. CP). On days −9, 56, and 111 none of the horses showed lameness (AAEP score 0). On days 0 and 14, an AAEP lameness score of 1 was observed in both forelimbs of all horses (Figure 2), indicating a bilateral lameness in the horses.

### Ultrasound Assessment

#### Echogenicity

**Figure 3:**
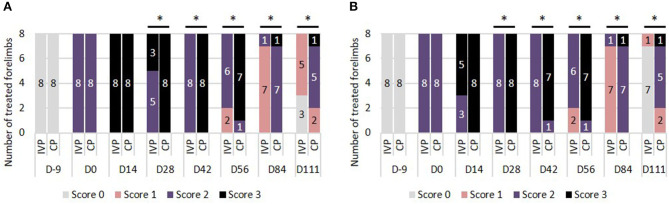
Ultrasound assessment. **(A)** Echogenicity scores: 0 = normal; 1 = mildly hypoechoic; 2 = moderately hypoechoic; 3 = severely hypoechoic. **(B)** Fiber alignment scores: 0 = ≥ 75%; 1 = 50–74%; 2 = 25–49%; 3 = ≤ 24%. *P-values ≤ 0.05.

There was no difference in echogenicity between the two treatment groups from day −9 until day 28. From day 28 onwards, the frequency of scores indicating hypoechoicity (score 1–3) was significantly lower in the IVP treated limbs in comparison to the CP treated limbs (*P* = 0.009 on day 28, *P* < 0.001 from day 42 to 84, *P* = 0.003 on day 111) (Figure 3A).

#### Fiber Alignment Score

There was no significant difference in fiber alignment score between the two groups from day −9 until day 28. From day 28 onwards, the percentage of parallel fiber bundles was significantly higher in the IVP group in comparison to the CP group (*P* < 0.001 at all time points) (Figure 3B).

**Figure 4:**
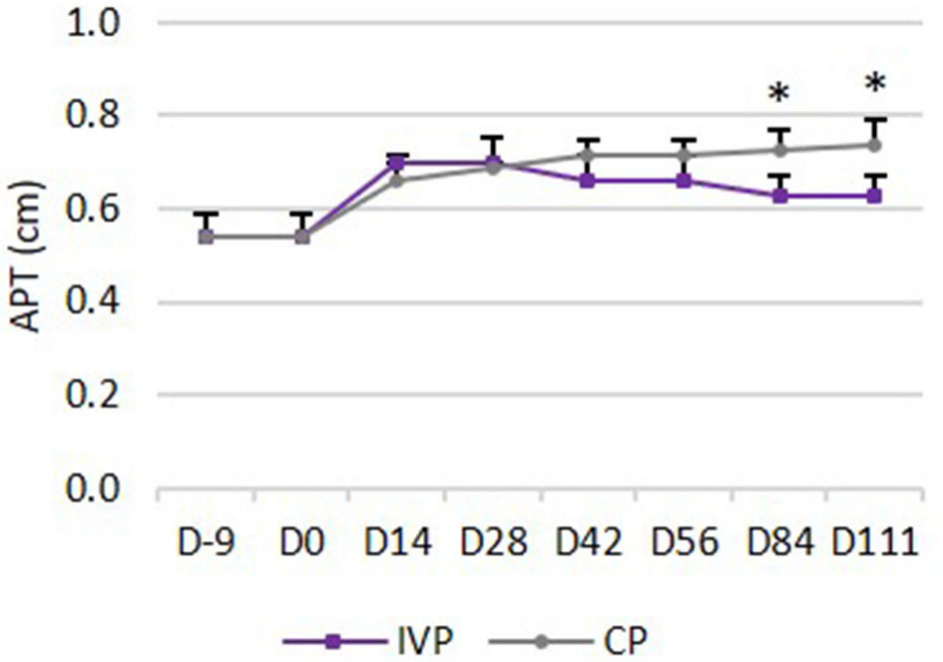
Mean (+SD) anterior-posterior thickness (cm). *P-values ≤ 0.05.

No significant differences could be observed in *anterior-posterior thickness (APT)* between both treatment groups from day −9 until day 84. The increase in APT was significantly lower of the IVP treated limbs compared to the CP treated limbs on day 84 (*P* = 0.010) and day 111 (*P* = 0.009) (Figure 4).

### UTC Assessment

**Figure 5:**
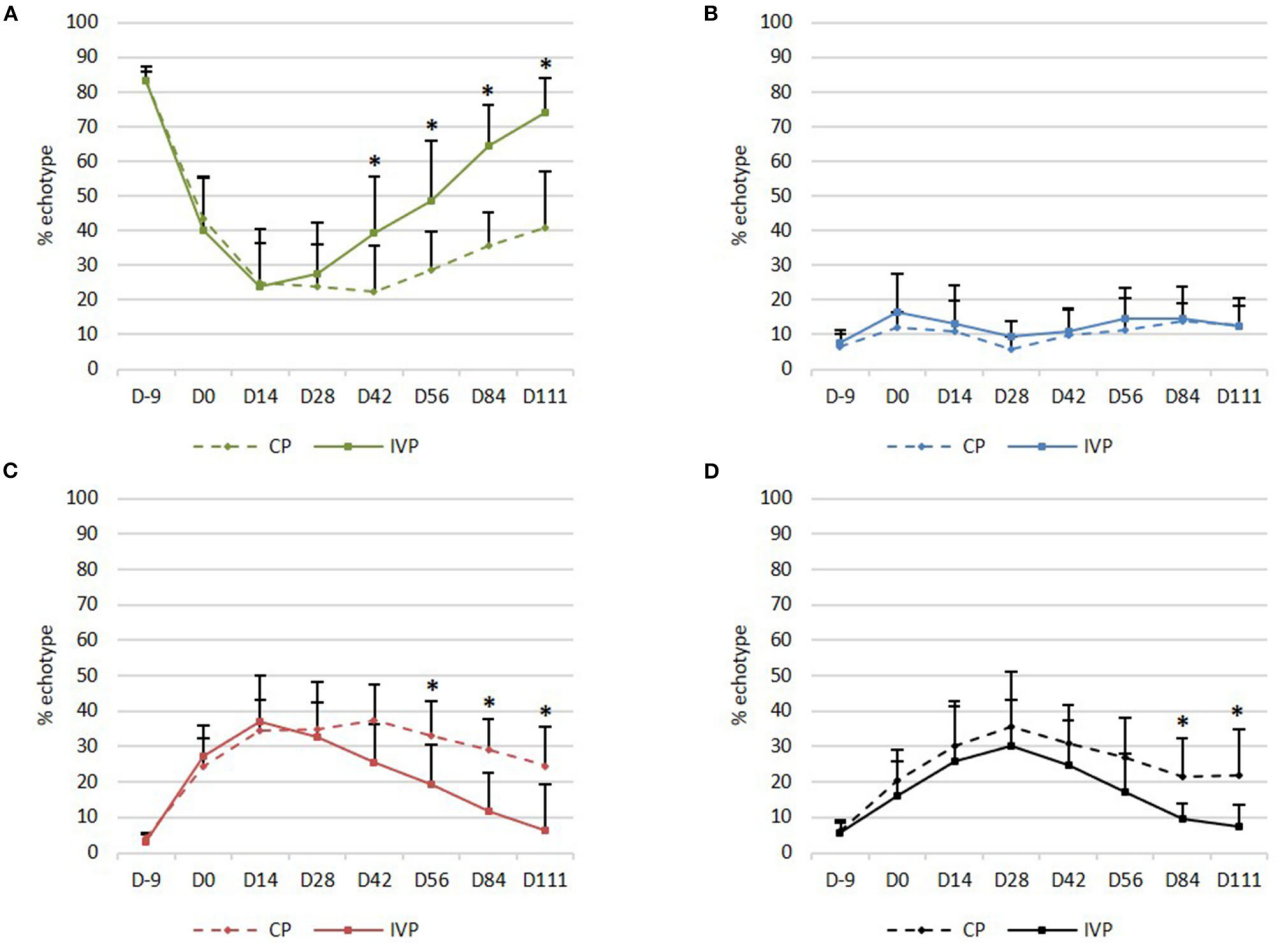
UTC assessment. **(A)** Mean (+SD) echo type I (green, fully aligned fascicles); **(B)** Mean (+SD) echo type II (blue, discontinuous and less aligned fascicles); **(C)** Mean (+SD) echo type III (red, fibrillar matrix); **(D)** Mean (+SD) echo type IV (black, amorphous matrix and fluid). *P-values ≤ 0.05.

*Echo type I* (echo generated by intact and fully aligned fascicles, green) was observed in the majority (82–84%) of the limbs in all treatment groups on day −9 (before surgery). This percentage dropped to 12–45% between day 0 and day 28. Significant differences between groups were seen from day 42 onwards, with significantly higher percentages for IVP treated limbs in comparison to the CP treated limbs (*P* = 0.039 on day 42, *P* = 0.026 on day 56, *P* = 0.009 on day 84, and *P* = 0.007 on day 111) (Figure 5A).

Percentage in *echo type II* (echo generated by discontinuous and less aligned fascicles, blue) was not significantly different between the two treatment groups at any observation day (Figure 5B).

*Echo type III* (echo generated by a mainly fibrillar matrix, red) was observed on day −9 in a relatively low percentage (3–5%) of the limbs in all treatment groups, whereas this percentage increased to 22–45% between day 0 and day 42. Significant differences between groups were seen from day 56 onwards, with lower percentages for IVP treated limbs when compared to the CP treated limbs (*P* = 0.032 on day 56, *P* = 0.009 on day 84 and *P* = 0.007 on day 111) (Figure 5C).

*Echo type IV* (echo generated by an amorphous matrix and fluid, black) was observed on day −9 in a relatively low percentage (6–7%) of the limbs in all treatment groups, whereas this percentage increased to 11–44% between day 0 and day 56. Significant differences between treatment groups were seen from day 84 onwards, with lower percentages for IVP treated limbs in comparison to the CP treated limbs (*P* = 0.026 on day 84 and *P* = 0.021 on day 111) (Figure 5D).

On day 111, a significantly higher percentage in echo type I (*P* = 0.008) and a significantly lower percentage in echo type III and IV (*P* = 0.008 and *P* = 0.016 respectively) was observed in the IVP treated limbs, as compared to day 0. No significant changes in percentage of each echo type were observed in CP treated limbs when compared to day 0.

Moreover, no significant differences could be observed in the IVP treated limbs between day −9 and day 111 for any echo type. This indicates that the tendon quality on day 111 after intralesional tpMSC treatment is comparable with the healthy tendon quality and structure as observed on day −9. This was not the case for the CP treated limbs, where significantly lower echo types I (*P* = 0.008) and significantly higher echo types III and IV (*P* = 0.008) were generated on day 111 compared to day −9.

### Macroscopic and Histopathologic Examination

#### Macroscopic Evaluation

**Figure 6:**
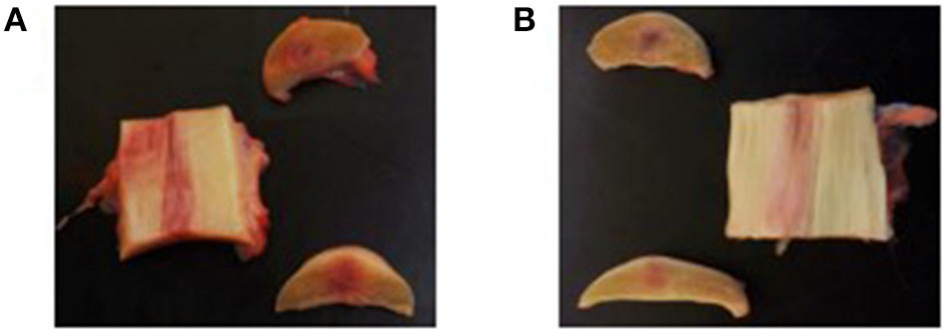
Macroscopic evaluation of the tendon tissue on day 112. **(A)** Longitudinal and transverse sections of the control product treated forelimb. **(B)** Longitudinal and transverse sections of the investigational product treated forelimb.

The lesion site was still visible in all SDFTs collected from both treatment groups, as well as discoloration of the tendon tissue (Figure 6). The thickness of the paratenon as well as the thickness of the curial lymph nodes was considered to be normal in all limbs of both groups.

#### Ectopic Tissue

No ectopic tissue was detected during histopathological examination of the tendon, paratenon, or the cubital lymph nodes.

#### Histologic Evaluation

In all organ and lymph node slides ectopic tissue and inflammation were not observed.

#### Scoring of the Tendon Tissue

No significant differences of fiber structure, fiber arrangement, roundness of the nuclei, regional variations in cellularity, vascularity, collagen stainability, glycosaminoglycan content and the presence of inflammatory cells could be observed between treatment groups (Table 7).

**Table 7:** Histological scoring of the tendon tissue.

**Parameter**	**IVP [mean (±SD)]**	**CP [mean (±SD)]**
Fiber structure	0.6 (0.9)	0.0 (0.0)
Fiber arrangement	0.6 (0.9)	0.1 (0.4)
Roundness of the nuclei	1.6 (0.9)	1.3 (0.5)
Regional variations in cellularity	1.6 (0.9)	1.9 (0.8)
Vascularity	1.8 (0.5)	1.4 (0.5)
Collagen stainability	2.1 (0.8)	1.6 (0.9)
Glycosaminoglycan content	2.8 (0.5)	2.4 (0.7)
Presence of inflammatory cells	0.6 (1.1)	0.5 (1.1)

*Score 0, normal; score 1, slightly abnormal; score 2, moderately abnormal; score 3, markedly abnormal*.

### Immunohistochemistry

**Figure 7:**
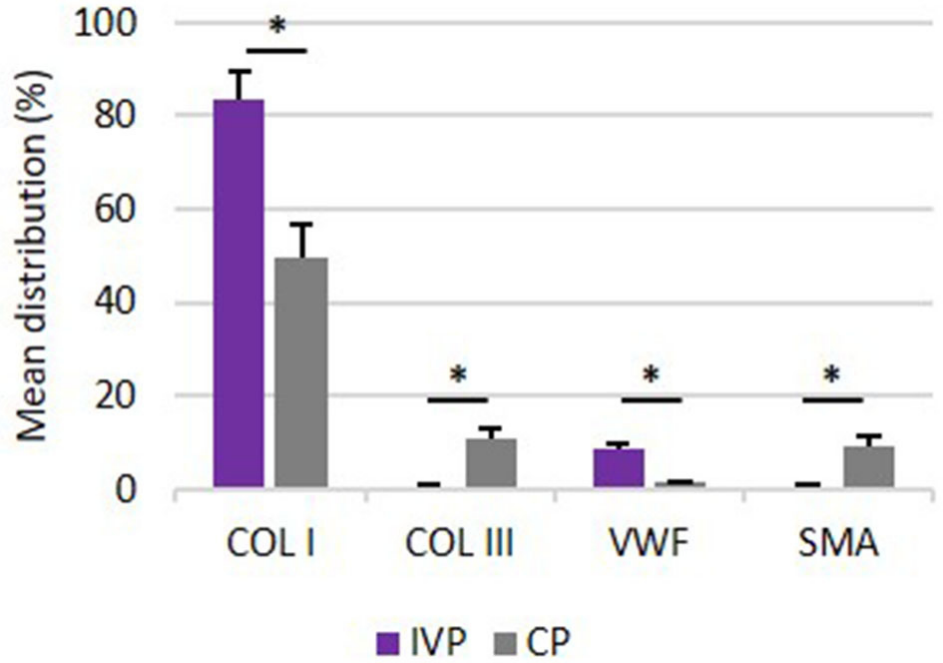
Percentage distribution of COL I, COL III, VWF and SMA. Comparison of mean distribution percentages (+SD) of extracellular matrix proteins between investigation product treated limbs (IVP) and control product treated limbs (CP). *P-values ≤ 0.05.

**Figure 8:**
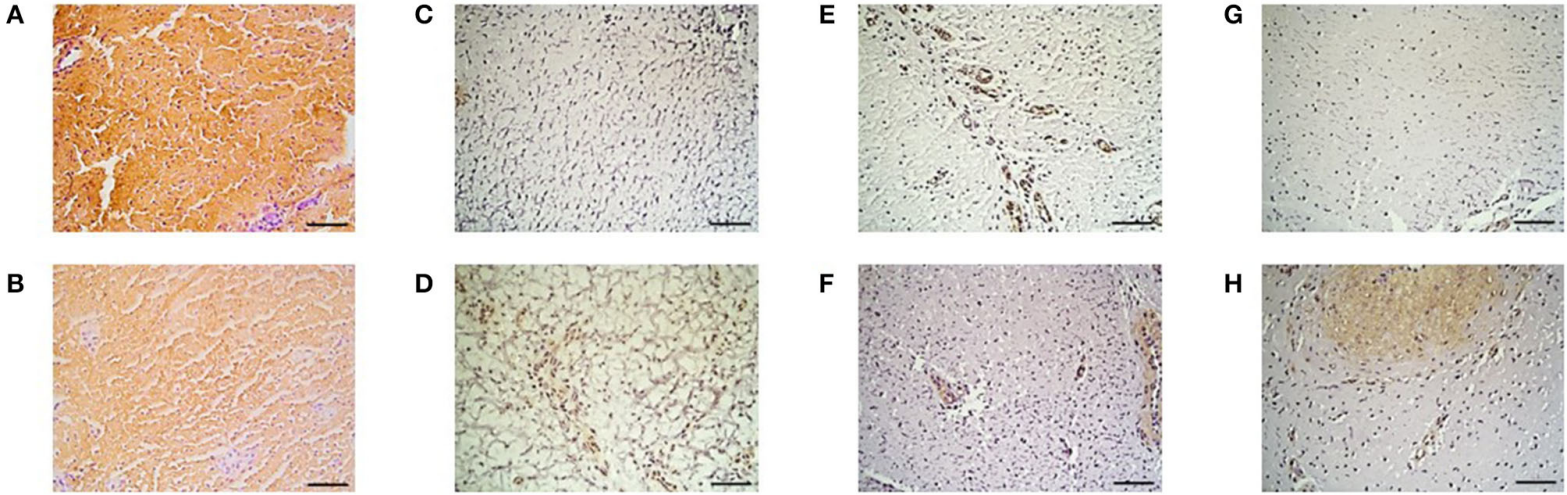
Immunohistochemistry. Collagen type I in the **(A)** IVP and **(B)** CP treated limb, collagen type III in the **(C)** IVP and **(D)** CP treated limb, Von Willebrand Factor in the **(E)** IVP and **(F)** CP treated limb and of smooth muscle actin in the **(G)** IVP and **(H)** CP treated limb. Scale bar = 10 μm.

The results of percent distribution and immunohistochemical stainings are depicted in Figures 7, 8, respectively.

#### Collagen Type I

The percent distribution of COL I was significantly higher for all IVP treated limb in comparison to CP treated limbs (*P* = 0.005).

#### Collagen Type III

The percent distribution of COL III was significantly lower for all IVP treated limb in comparison to CP treated limbs (*P* = 0.005).

#### Von Willebrand Factor

VWF was significantly higher for all IVP treated limbs in comparison to CP treated limbs (*P* = 0.005).

#### Smooth Muscle Actin

The percent of distribution of SMA was significantly lower for all IVP treated limb in comparison to CP treated limbs (*P* = 0.005).

## Discussion

This randomized, blinded and placebo-controlled study was performed to evaluate the safety, tolerance, and efficacy of an intralesional injection with allogeneic equine peripheral blood-derived tenogenic primed mesenchymal stem cells (tpMSCs) on surgically induced SDFT core lesions. The adapted surgical procedure from Schramme et al. (31) was performed in the SDFT of both forelimbs of each horse. This model provides a more standardized and localized injury in comparison with enzymatically induced lesions (36) and resembles the naturally occurring injuries more closely in terms of clinical, ultrasonographic, and histological findings (33, 37). The surgical procedure induced a tendon lesion in both forelimb SDFT's, which could be confirmed by ultrasound and UTC assessment. Because fibroplasia generally begins around the seventh day after injury (29), the treatment was administered 1 week following surgery, after the acute phase of the inflammatory response caused by the surgical induction of the SDFT core lesion has subsided (38, 39). One forelimb of each horse received an intralesional injection with tpMSCs and the contralateral forelimb was treated with saline to serve as an intra-animal control. This design allows to minimize the variability in tendon quality between treatment groups, and the number of experimental animals used (21, 33). This study design was similar to a study by Bosch et al. (33) evaluating neovascularization of tendon tissue after the surgical induction of a SDFT core lesion. In that study evaluating six horses, one forelimb was treated with platelet-rich-plasma (PRP) and the contralateral limb with placebo (saline) to serve as an intra-animal control. Increased neovascularization in the PRP treated limbs could be demonstrated at 24 weeks post-injection, pointing out the relevance of the current study protocol and the number of horses used.

All eight treated horses were controlled for adverse events. Only one adverse event, a colic episode at New Year's Eve (38 days post-treatment), was observed in one horse, and the patient fully recovered the next day without the need for treatment. The adverse effect was not regarded to be related to the study medication since the incidence in this study (1 episode in 1 out of 8 horses over 121 days of observation) is similar to the incidence rate of colic in horses observed in the field (3.5–10.6 cases per 100 horse years) (40). No other serious adverse events or suspected drug reactions were observed throughout the study period.

No significant difference between the tpMSC and contralateral placebo treated limbs was observed for heat and pain to pressure. Swelling was observed after surgery in all treatment groups with similar distribution of scores. A significantly higher swelling in the IVP treated limbs was recorded on day 9 and 10, resulting in a higher tendon circumference in comparison to baseline values. This reaction might be related to the tpMSC treatment, however no other clinical signs were associated. The swelling was limited (2 mm), temporary (2 days) and decreased from day 11 onwards. Comparable studies also observed swelling following intra-lesional MSC treatment, reporting a decrease from week 2 or week 8 after MSC injection onwards using autologous BM-MSCs (41) and AT-MSCs (23, 42).

Lameness (AAEP score 1) was observed on days 0 and 14 in all forelimbs, but not at further lameness examinations. Within the first and second week after an acute SDFT injury, mild transient lameness can occur (43). Schramme et al. (31) reported mild to moderate lameness in the 6-day period after surgical induction of a bilateral SDFT core lesion. Within the following 2–7 days, lameness resolved rapidly. The observation of lameness on days 0 and 14 is likely related to the surgical procedure and is consistent with the clinical timeline observed with naturally occurring tendon lesions.

Ultrasonography is the standard method to assess tendon healing over time. Significant differences in fiber alignment score and echogenicity between groups could be observed from day 28 onwards. The frequency of scores indicating hypoechoicity and a relatively low percentage of parallel fiber bundles were significantly lower in tpMSC treated limbs in comparison to saline treated limbs. At the end of the study period, 7 out of 8 tpMSC treated limbs compared to 0 out of 8 control limbs showed over 75% parallel fiber bundles in the lesion (score 0). Additionally, hypoechoic structures persisted significantly longer in the placebo treated limbs compared to tpMSC treaded forelimbs. This is in line with a previously performed study where an improvement of the fiber alignment score was observed at 6 and 12 weeks after a combined tpMSC-PRP treatment (29). Dyson et al. (5) demonstrated that the fiber alignment score at 16 weeks can be correlated with the re-injury rate after 2 years. All horses with a fiber alignment score of 2 or 3 suffered from a recurrent injury of the treated limb, whereas no horse with a fiber alignment score of 0 re-injured the treated limb. This might indicate that the significantly better results of the tpMSC treatment in the current study in comparison to the placebo treatment may lower the risk of re-injury in the long-term. However, a field study should be performed in order to confirm this hypothesis.

On the other hand, it has been shown that conventional ultrasonography is not sufficiently sensitive to accurately and unequivocally evaluate the composition of tendon tissue (44). In a human study, a lower score for hypoechoicity was not correlated with an improved clinical outcome (45). Therefore, ultrasound tissue characterization (UTC) was included in the present study to improve the objective tendon characterization by standardizing instrumental settings. The UTC assessment showed significant differences between groups from day 42 onwards for echo type I. The tpMSC treated limbs showed a significantly higher echo type I percentage in comparison to the placebo group. This echo type corresponds to healthy tendon structure consisting of fully aligned intact tendon structures with a high amount of collagen type I fibers (32). The percentages of echo type III and echo type IV were significantly lower in the tpMSC treated tendons in comparison to the placebo group from day 56 to 84 onwards, respectively. Moreover, no significant differences could be observed in the IVP treated limbs between day −9 (before surgery) and day 111 for any echo type. This indicates that the tendon quality on day 111 after intralesional tpMSC treatment is comparable with the healthy tendon quality and structure as observed on day −9.These findings demonstrate that a more mature and superiorly healed tendon was present in the tpMSC treated group at the end of the study compared to the placebo group (32). The UTC results of the placebo group reported by Bosch et al. (32) at 16 weeks post-injection are in line with the placebo data of the current results 16 weeks after treatment for types I and IV. This demonstrates that the model as well as the UTC scans were performed in a similar way. However, when looking at PRP vs. tpMSC treatment considerable differences could be noticed at the same time point between the studies. Bosch et al. used a preliminary version of the tracker, which was moved by hand along a magnetic strip (window size 9), rather than the automatic tracker used here. The results for PRP in that study at week 16 showed much lower values of echo type I compared to tpMSC treatment in our study (56 vs. 74%). Approximately the same percentages for all echo types were reached at the end of the PRP study at week 24 as at week 16 in our study. This indicates a faster structural and functional healing of the tendons treated with tpMSCs in comparison to the PRP treatment.

The observations of the UTC assessment were confirmed by the immunohistochemistry results obtained on day 112. The high percentage of echo type I corresponding to healthy tendon structure with a high amount of collagen type I fibers are in line with the significantly higher distribution of collagen type I in the tpMSC treated limbs compared to the placebo treated limbs. Strength and elasticity of the tendon is provided by collagen type I as the major fibrillar component. The presence of a high amount of collagen type I is key for a functional, high-quality healing of the tendon tissue and an important indicator of tendon matrix synthesis (12, 21). In the placebo treated limbs, more fibrotic tissue was observed as represented by a higher percentage of echo type III on UTC and a higher distribution of collagen type III and SMA and a lower amount of blood vessels on immunohistochemistry. The SMA protein is incorporated in myofibroblasts that play a role in early post-inflammatory events after tendon injury (11). An increased number of myofibroblasts results in the synthesis of abundant amounts of COL III, resulting in the formation of persistent scar tissue (46–48), reduced blood flow and eventually chronic tendinopathies (49). The poor vascularization of tendons is considered to be one of the reasons for their limited healing potential (33). Blood vessels can be visualized by staining for VWF, which can be found in functional endothelial cells and thus acts as a marker for vascularization (50). The significantly higher percentage of VWF in the tpMSC treated limbs in the current study indicates a significantly enhanced healing capacity in these limbs (33). In a similar study performed by Conze et al. (50), where nine horses received AT-MSCs in a surgically created SDFT lesion in one forelimb and a placebo (inactivated autologous serum) in the contralateral forelimb, an increased neovascularization of the AT-MSC treated tendons was also observed at 22 weeks post-treatment. In the present study no differences could be detected between both treatment groups on histological sections, resulting in an inconsistency with the UTC and immunohistochemistry data. Although some studies could identify differences in standard histopathological parameters (20, 51), other studies only use immunohistochemistry instead of standard histopathology to obtain objectively quantified results (33). The absence of differences between the IVP and the CP treated tendons could be due to the fact that only one section of tissue was imaged for histological evaluation.

Another limitation of this study was including both forelimbs making lameness harder to interpret, although only mild lameness was observed during the study period without scoring difficulties. Furthermore, the tpMSCs were not labeled so no conclusions could be drawn in the matter of cell survival and retention. Cell labeling was not performed to avoid alterations of the final IVP that might influence the mode of action. There was no biomechanical testing data or return to exercise data to investigate the risk of re-injury. Additionally, an experimental model was used to obtain standardized circumstances. However, naturally occurring tendon injuries present more variability and cannot always be treated on the right time point in the healing process. Therefore, the results of this study should be confirmed in a field trial on a large number on horses suffering from naturally occurring tendon disease.

This study demonstrates that allogeneic tpMSCs are well-tolerated and may be effective in the management of tendon injuries in horses. A higher percentage of intact and fully aligned fascicles on UTC in the tpMSC treated limbs in comparison to placebo treated limbs demonstrate the beneficial effects of tpMSCs for the treatment of tendon injuries in horses.

## Conclusion

The administration of tenogenic primed mesenchymal stem cells resulted in significantly higher amounts of intact and fully aligned fascicles by UTC assessments and collagen type I distribution by immunohistochemistry compared to a placebo treatment after a single intralesional injection in surgically created SDFT core lesions. Additionally, significantly improved echogenicity scores and fiber alignment scores were recorded. Therefore, equine allogeneic tpMSCs are a promising therapeutic for tendon injuries in horses.

## Data Availability Statement

The original contributions presented in the study are included in the article/Supplementary Material, further inquiries can be directed to the corresponding author.

## Ethics Statement

The animal study was reviewed and approved by an independent ethics committee approved by the Flemish Government (permit number: LA1700607).

## Author Contributions

JS, SB, and LVH conceived the study and planned the design. Daily clinical assessments and periodical tendon assessments of the horses were performed by SB. HVS provided the tools and knowledge of the ultrasonographic tissue characterization. Ultrasound and ultrasound tissue characterization assessments were performed by SB and ED. The surgical induction of the core lesion was performed by AM and the treatment administration was done by FP. KC, LVB, and SB performed full necropsy and histopathology. ED wrote the first draft of the manuscript. JS, SB, CB, LVH, AM, FP, KC, LVB, and HVS provided supervision and critical review of the manuscript. All authors contributed and approved the final version of the manuscript.

## Conflict of Interest

JS declares competing financial interests as shareholder in GST at the time of the study. SB, JS, ED, CB, and LH were all employed by GST at the time of the study. The content of this manuscript contains a stem cell product under development (Tendo-Cell® Plus) owned by GST and patented under the following numbers: EP13799605.4, CA2928122, US15/038,172. The remaining authors declare that the research was conducted in the absence of any commercial or financial relationships that could be construed as a potential conflict of interest.

## Abbreviations

AAEP: American association of equine practitioners
APT: Anterior-posterior thickness
AT-MSC(s): Adipose tissue-derived mesenchymal stem cell(s)
BM-MSC(s): Bone marrow-derived mesenchymal stem cell(s)
COL I: Collagen type I
COL III: Collagen type III
CP: Control product
DAB: Diaminobenzidine
EDTA: Ethylenediaminetetraacetic acid
HRP: Horseradish peroxidase
IVP: Investigational product
MSC(s): Mesenchymal stem cell(s)
PBS: Phosphate buffered saline
PRP: Platelet rich plasma
SDFT: Superficial digital flexor tendon
SMA: Smooth muscle actin
tpMSC(s): Tenogenic primed mesenchymal stem cell(s)
UTC: Ultrasound tissue characterization
VWF: Von Willebrand factor.
