# Supplementary material for: The Evaluation of Equine Allogeneic Tenogenic Primed Mesenchymal Stem Cells in a Surgically Induced Superficial Digital Flexor Tendon Lesion Model
Source: Front Vet Sci. 2021 Mar 5;8:641441. doi: 10.3389/fvets.2021.641441 (PMC7973085; doi:10.3389/fvets.2021.641441)
Supplement: Supplementary file 1 [file Table_1.pdf]

Supplementary Table 1. Mean (± SD) hematological and serum biochemistry results on day -10, day 0, day 14 and day 111.

	**Day -10 Mean (± SD)**	**Day 0 Mean (± SD)**	**Day 14 Mean (± SD)**	**Day 111 Mean (± SD)**
Red blood cells (10^12/L)	8.88 (± 1.1)	8.95 (± 0.6)	8.46 (± 1.4)	8.83 (± 1.2)
White blood cells (10^9/L)	7.88 (± 1.0)	8.11 (± 0.9)	7.00 (± 2.3)	6.38 (± 0.9)
Lymphocytes (10^9/L)	2.78 (± 0.8)	2.81 (± 0.7)	2.69 (± 0.9)	2.83 (± 0.8)
Monocytes (10^9/L)	0.36 (± 0.1)	0.40 (± 0.1)	0.32 (± 0.2)	0.27 (± 0.1)
Eosinophils (10^9/L)	0.64 (± 0.6)	0.53 (± 0.5)	0.48 (± 0.5)	0.22 (± 0.2)
Neutrophils (10^9/L)	4.05 (± 0.7)	3.89 (± 0.5)	3.44 (± 2.0)	3.03 (± 0.5)
Basophils (10^9/L)	0.12 (± 0.1)	0.18 (± 0.1)	0.07 (± 0.0)	0.04 (± 0.0)
Platelet count (10^9/L)	143.13 (± 49.1)	138.88 (± 33.7)	123.63 (± 38.7)	122.50 (± 38.9)
Reticulocytes (/µL)	6000.00 (± 2289.6)	4685.00 (± 704.7)	5960.00 (± 2257.2)	10070.00 (± 4707.7)
Hematocrit (L/L)	0.42 (± 0.1)	0.43 (± 0.0)	0.41 (± 0.1)	0.43 (± 0.1)
Hemoglobin (g/dL)	14.08 (± 1.8)	14.44 (± 1.3)	13.55 (± 2.3)	14.31 (± 1.9)
Globulin (g/L)	70.63 (± 106.4)	33.88 (± 9.5)	32.88 (± 13.7)	29.75 (± 9.2)
Mean corpuscular hemoglobin (pg)	16.00 (± 0.9)	16.25 (± 1.2)	16.00 (± 0.9)	16.25 (± 0.7)
Mean corpuscular hemoglobin concentration (g/dL)	33.25 (± 0.5)	33.75 (± 1.0)	33.38 (± 0.5)	33.50 (± 0.5)
Mean corpuscular volume (fl)	47.63 (±2.7)	47.50 (± 2.4)	48.25 (± 2.5)	48.75 (± 2.4)
Albumin (g/L)	34.75 (± 2.5)	34.38 (± 3.2)	33.88 (± 3.6)	34.13 (± 1.9)
Alkaline phosphatase (IU/L)	240.25 (± 134.3)	213.88 (± 106.1)	179.63 (± 69.2)	127.50 (± 47.5)
Aspartate aminotransferase (IU/L)	569.50 (± 275.4)	655.00 (± 478.2)	655.88 (± 348.0)	323.25 (± 45.5)
Creatinine kinase (IU/L)	284.63 (± 68.5)	219.38 (± 55.4)	239.38 (± 78.8)	235.38 (± 51.2)
Lactate dehydrogenase (IU/L)	369.00 (± 111.4)	350.13 (± 94.2)	361.25 (± 176.5)	293.13 (± 76.5)
Blood urea nitrogen (mg/dL)	12.25 (± 2.4)	16.38 (± 2.2)	14.38 (± 1.9)	14.50 (± 2.0)
Creatinine (mg/L)	12.63 (± 1.6)	12.38 (± 1.8)	10.75 (± 1.5)	11.25 (± 2.3)
Direct bilirubin (mg/L)	4.71 (± 1.3)	4.33 (± 0.9)	4.01 (± 1.0)	4.05 (± 0.7)
Gamma-glutamyl transferase (IU/L)	63.13 (± 49.4)	53.00 (± 37.0)	56.00 (± 25.9)	32.75 (± 16.2)
Total bilirubin (mg/L)	15.38 (± 4.4)	17.01 (± 6.3)	15.00 (± 5.5)	21.65 (± 6.0)
Total protein (g/L)	67.50 (± 7.6)	68.25 (± 7.8)	66.75 (± 11.0)	63.88 (± 8.1)
Urea (g/L)	0.27 (± 0.1)	0.35 (± 0.0)	0.31 (± 0.0)	0.31 (± 0.0)
